# Targeting Ribosome Biogenesis in Cancer: Lessons Learned and Way Forward

**DOI:** 10.3390/cancers14092126

**Published:** 2022-04-24

**Authors:** Asimina Zisi, Jiri Bartek, Mikael S. Lindström

**Affiliations:** 1 Division of Genome Biology, Department of Medical Biochemistry and Biophysics, Karolinska Institutet, SciLifeLab, S-171 21 Stockholm, Sweden; asimina.zisi@ki.se (A.Z.); jb@cancer.dk (J.B.); 2 Danish Cancer Society Research Center, DK-2100 Copenhagen, Denmark

**Keywords:** ribosome biogenesis, nucleolus, p53, cancer, RNA polymerase I, translation

## Abstract

**Simple Summary:**

Cells need to produce ribosomes to sustain continuous proliferation and expand in numbers, a feature that is even more prominent in uncontrollably proliferating cancer cells. Certain cancer cell types are expected to depend more on ribosome biogenesis based on their genetic background, and this potential vulnerability can be exploited in designing effective, targeted cancer therapies. This review provides information on anti-cancer molecules that target the ribosome biogenesis machinery and indicates avenues for future research.

**Abstract:**

Rapid growth and unrestrained proliferation is a hallmark of many cancers. To accomplish this, cancer cells re-wire and increase their biosynthetic and metabolic activities, including ribosome biogenesis (RiBi), a complex, highly energy-consuming process. Several chemotherapeutic agents used in the clinic impair this process by interfering with the transcription of ribosomal RNA (rRNA) in the nucleolus through the blockade of RNA polymerase I or by limiting the nucleotide building blocks of RNA, thereby ultimately preventing the synthesis of new ribosomes. Perturbations in RiBi activate nucleolar stress response pathways, including those controlled by p53. While compounds such as actinomycin D and oxaliplatin effectively disrupt RiBi, there is an ongoing effort to improve the specificity further and find new potent RiBi-targeting compounds with improved pharmacological characteristics. A few recently identified inhibitors have also become popular as research tools, facilitating our advances in understanding RiBi. Here we provide a comprehensive overview of the various compounds targeting RiBi, their mechanism of action, and potential use in cancer therapy. We discuss screening strategies, drug repurposing, and common problems with compound specificity and mechanisms of action. Finally, emerging paths to discovery and avenues for the development of potential biomarkers predictive of therapeutic outcomes across cancer subtypes are also presented.

## 1. Introduction

The past 20 years have provided us detailed knowledge about how various cancers develop at the cellular and molecular levels. We have also seen the emergence of novel cancer treatment options, including tailor-made small molecules and immunotherapies. However, cancer therapy still largely depends on standard treatment modalities, including conventional chemotherapy, surgery, and radiation. In parallel with the development of precision oncology, there are efforts to target cancer cells from a slightly broader angle. In focus are the acquired hallmarks of cancer cells, including but not limited to unrestrained growth and proliferation, metastasis, and angiogenesis [1]. One such line of research aims at inhibiting the cancer cell’s production of new ribosomes. Ribosome biogenesis (henceforth denoted RiBi) is a fundamental multistep cellular process through which ribosomes, the cell’s protein factories, are built.

To briefly recapitulate the RiBi mechanism, three DNA-dependent RNA polymerases, ~80 ribosomal proteins (RPs), and a transient incorporation of approximately 200 non-ribosomal factors are utilized in the process [2]. The rate-limiting step is considered the transcription of ribosomal DNA (rDNA) into ribosomal RNA (rRNA) by RNA polymerase I (Pol I); rDNA transcription itself starts when the pre-initiation complex (PIC) is assembled at the rDNA promoter [2,3,4]. PIC formation requires binding of at least three transcription factors, the transcription initiation factor I (TIF-I), the upstream binding factor (UBF), and the promoter selectivity factor (SL1). Upon UBF binding to DNA, a nucleosome-like structure is formed that recruits Pol I and multiple Pol I-associated factors, forming a multiprotein complex termed the Pol I holo-complex [3,4,5]. The active rDNA genes are transcribed into the 47S rRNA precursor (47S pre-rRNA), which following further processing (cleavage and modification) forms the 18S, 5.8S, and 28S mature rRNAs. RNA Polymerase II (Pol II), in turn, transcribes the mRNAs of the RPs, while RNA Polymerase III (Pol III) transcribes the 5S rRNA in the nucleoplasm [6]. Mature 5S rRNA and RPs are translocated to the nucleolus and assembled with the other rRNAs to form the large and small ribosomal subunits, which will subsequently form the mature ribosomes after translocation to the cytoplasm and further modifications. Important to realize are the essential roles of all three RNA polymerases, and that failure of any one of these eventually leads to downregulation of RiBi.

The complexity of RiBi provides several opportunities to block any of the steps involved. One of the most clear-cut strategies to target RiBi is the inhibition of Pol I; such an inhibitor could effectively constrain aberrant or elevated rDNA transcription [7]. Indeed, the emergence of chemical RiBi inhibition as an anti-cancer therapeutic strategy has enabled the discovery of small-molecule rRNA transcription inhibitors as well as the functional assessment of clinically approved compounds that may be repurposed on the basis of their RiBi-inhibitory potential [7,8,9,10,11,12]. In this review, we aim to comprehensively list these compounds and their status in pre-, and clinical development. We highlight promising findings that can be further developed from a drug discovery perspective and discuss some problematic issues and questions that remain to be solved.

## 2. Ribosome Biogenesis as a Target in Cancer Cells

Why focus on targeting RiBi in cancer cells? There are, as we see it, three main arguments or cornerstones that support this concept. First, RiBi is a highly active and essential process in certain cancer types and cancer cell populations. Second, several commonly used and successful chemotherapy drugs are already known to partly exert their pharmacological effects by impairing RiBi. Third, blocking RiBi leads in many cases to the activation of the cell’s guardian protein, p53. The background to these cornerstones follows in greater detail below.

### 2.1. Ribosome Biogenesis Is Often Increased in Cancer Cells

Cancer cells are considered to have unlimited replicative potential, the ability to more frequently divide, and they often display higher rates of biosynthesis and overall metabolic activity [1]. Fast dividing cancer cells require enhanced global protein synthesis. The synthesis of proteins can be controlled in different ways, for example, by transcribing more mRNA or through an increase in mRNA translation [13]. The oncogene c-MYC is a key driver of cancer cell growth, and it helps boost transcription- and translation-related processes; thus, c-MYC is thought to drive RiBi [14,15,16,17]. The concept of “ribosomes translating cancer” has become particularly popular [18]. Besides the translation of mRNAs, there is a need to increase RiBi, and this represents the first cornerstone.

What is the evidence that RiBi is increased in cancer cells when compared to normal cells? RiBi is carried out mostly in the sub-nuclear, membrane-less compartment known as the nucleolus; the nucleoli emerge at the sites of actively transcribed tandem arrayed copies of rRNA genes, the nucleolar organizer regions [19]. The size and shape of the nucleolus may directly be related to the rDNA transcription rate and, as suggested in some studies, to the tumor lesion’s degree of malignancy, revealing its significance in pathology [20]. Indeed, pathologists have been paying attention to nucleolar size and morphology for over a century [21]. Some caveats apply; there are rapidly proliferating normal cells, for example, in the hematopoietic system and the epithelium of the colon. The opposite also holds true; some populations of cancer cells are relatively quiescent, such as the more elusive therapy-resistant cancer stem cells that may require additional targeting strategies. Nonetheless, normal cells are in general considered to have lower RiBi rates than their malignant counterparts, thereby opening a therapeutic window.

How is RiBi activity enhanced in the rapidly growing cancer cell? Multiple signaling pathways converge on the RNA Pol I machinery and the nucleolus, making it a dynamic structure and a sensory hub of internal and external cellular stimuli [22,23,24,25,26,27]. This regulatory connection assigns the nucleolus with several direct or indirect functional roles, including regulation of cell cycle progression, cell growth, and cellular stress responses [27].

Oncogenic pathways have been identified that regulate Pol I activity to enhance rDNA transcription [3]. The various signalling cascades often result in activated phosphatidylinositol 3-kinase and Protein Kinase B pathways, which in turn converge on c-MYC and the mammalian target of rapamycin (mTOR) pathway. c-MYC mediates SL1 recruitment to promoters via direct interaction with rDNA loci inducing Pol I transcription [16,28,29]. Moreover, c-MYC promotes the synthesis of RPs through stimulation of Pol II transcription and activates the Pol III transcription factor TFIIIB, enhancing Pol III activity and the synthesis of 5S rRNA [15,17]. On the other hand, mTOR activates Pol I via phosphorylation of factor TIF-IA and Pol III through the interference of TFIIIB and TFIIIC with 5S rRNA [30,31]. Additionally, positive stimuli can activate RAS-MAPK (Mitogen-activated protein kinase), causing post-translational modifications such as phosphorylation of UBF, SL1, and TIF-IA, boosting rDNA transcription [4].

In contrast, the Pol I machinery is negatively regulated by tumor suppressors frequently mutated or lost in cancers [11,32]. For example, p53 dampens Pol I activity through its interaction with SL1, hindering PIC assembly at the rDNA gene promoters; p53 also restrains Pol III activity by directly binding to TFIIIB [4]. Other tumor suppressor proteins that put the brakes on RiBi include the retinoblastoma protein (RB), p14/p19ARF, and phosphatase and tensin homolog (PTEN) [3]. Taken together, the connection of Pol I activity to oncogenic and tumor-suppressing pathways commonly deregulated in cancer often results in enhanced RiBi rates, rendering it a relevant target for the rational design of cancer cell-selective small-molecule inhibitors.

### 2.2. Chemotherapy Often Targets Ribosome Biogenesis

The second cornerstone reflects the finding that several traditional and highly effective chemotherapy agents inhibit RiBi. This is probably not a coincidence. To begin with, most of the drugs used in standard-of-care cancer chemotherapy fall into the following chemical groups: (i) the DNA intercalators and minor groove binders, consisting of synthetic compounds and natural antibiotics with planar aromatic systems, (ii) the cross-linking and alkylating agents, which target DNA by forming DNA adducts, (iii) the antimetabolites, analogs of cellular metabolites that interfere mainly with nucleic acid synthesis, (iv) the plant-derived alkaloids, a diverse group, featuring among others, topoisomerase inhibitors, and (v) non-intercalating antibiotics with diverse pharmacological effects. Several chemotherapeutic agents activate the DNA damage response (DDR). The DDR employs a network of checkpoint kinases including DNA-PK, ATM, ATR, CHK1, CHK2, and MAPK activated protein kinase 2 (MK2) that mediate DNA damage signalling and contribute to p53 activation [19]. p53, in turn, executes programs of cell-cycle arrest, DNA damage repair, autophagy, senescence, or apoptosis [33]. However, the DDR is often dysfunctional in cancer cells, sometimes making them more vulnerable, and others more resistant. On the downside, chemotherapeutics may have genotoxic effects in normal cells as well, causing both short- and long-term side effects. Moreover, treatment may result in the development of drug resistance and an increased risk of tumor recurrence.

Chemotherapeutic agents often elicit pleiotropic pharmacological effects, and despite their extensive clinical use, the molecular targets are still not fully deciphered. Burger et al. elegantly demonstrated how several common chemotherapeutics impair RiBi at various steps [34]. Among these are alkylating agents, anti-metabolites (5-FU), alkaloids, and inhibitors of topoisomerases. While the available evidence intuitively supports RiBi as an important target of chemotherapeutic agents, the overall picture appears more complex and can be challenged [35]. For example, in the study by Burger [34], it was not shown whether the observed effects on RiBi can be connected to cell death and whether the effect represents the compounds’ primary mechanism of action. In other words, to what extent the blockade of RiBi contributes to the cytotoxic effects and overall clinical efficacy needs a careful evaluation for each specific compound

### 2.3. Ribosome Biogenesis Dysfunction Often Leads to p53 Activation

The third cornerstone, which is of central importance, is the intimate connection between RiBi and p53. It is well established that the p53 transcription factor is activated in response to numerous cellular damage/stress signals, such as DNA damage, oncogenic activation, hypoxia, or other insults to critical cellular functions [33]. At the same time, impaired RiBi is seen in response to a broad range of insults, including certain nutrient deprivation, changes in redox balance, DNA damage, hypoxia, or mutations affecting diverse nucleolar proteins [22,36,37]. Functional defects in the RiBi process trigger the ribosomal stress response, also called nucleolar stress, one of the key surveillance pathways of the cell, leading to p53 stabilization [38,39,40,41,42,43].

But how is p53 activated by RiBi inhibition? More than 20 years ago, it was shown that liver cells that cannot produce new ribosomes due to the inactivation of ribosomal protein S6 fail to divide due to activation of a cellular checkpoint that arrests the cell cycle [44]. Later, p53 activity was found to be increased following the expression of a mutant nucleolar protein, Bop1, that is involved in rRNA processing [45]. In this study, the first reference to nucleolar stress is found. While mouse double minute 2 protein (MDM2) was linked to ribosomal RNA and RPL5 in 1994 [46], the importance of this connection did not really become more apparent until ten years later. It turns out that p53 is activated when a pre-ribosome assembly complex known as 5S RNP increases in abundance. It tends to increase in free form when RiBi does not function properly or is in overdrive upon oncogenic c-MYC activity [47,48,49]. Upon deficiency in RiBi, the ribosomal proteins RPL5 and RPL11, together with the 5S rRNA, form the 5S RNP complex that, in turn, interacts with and sequesters the MDM2–an E3 ubiquitin ligase–which under normal conditions drives the proteasomal degradation of p53; thus this sequestration of MDM2 by 5S RNP leads to stabilization of the p53 tumor suppressor [47,48,50,51,52]. Herein we will use the impaired ribosome biogenesis checkpoint (IRBC) with reference to the events that induce this complex and its binding to MDM2. Chemotherapeutic agents disrupting RiBi and the nucleolus often cause the stabilization of p53 [53]. However, there are also compounds that impair RiBi (presumably indirectly), yet stabilize and/or activate p53 through other pathways than IRBC, for example by blocking proteasomal degradation of p53 or by affecting p53 post-translational modifications [34,54,55].

The p53 pathway is often inactivated by mutations, alterations in MDM2, or other factors [56,57,58]. Despite this caveat about p53, there are reasons for optimism, because downregulation of RiBi in p53 mutant or null cells also leads to impaired cell growth. p53-independent mechanisms of sensing and signaling nucleolar stress have been reviewed [36,59]. One potentially important mechanism involves enhanced RPS14 binding to cyclin-dependent kinase 4 (CDK4) in senescent cells, thereby preventing phosphorylation of RB [60,61]. Moreover, in response to nucleolar stress, degradation of the E2F-1 transcription factor is observed [62]. There are efforts underway to restore the function of mutant p53 or re-introduce wild type p53, and such strategies may be used to complement or potentiate the RiBi stress-inducing agents [63]. In summary, that different cancer drugs often inhibit RiBi, a highly active process in several cancer types, and that p53 is so intimately linked to this process together justify a strong case in favor of the continued focus on RiBi as a promising target in cancer therapy.

## 3. Clinically Approved Drugs and Their Effect on RiBi

In this section, we will describe clinically approved compounds that have been shown to interfere with RiBi. We will also revisit a few published drug repurposing studies, aiming to further highlight clinically used compounds for their potential to target RiBi. Drug repurposing can significantly speed up the process of clinical trials, assigning old drugs to new indications, commonly at a lower cost.

### 3.1. DNA Intercalators

One of the best-studied rRNA synthesis inhibitors is actinomycin D or dactinomycin (ActD for short), a natural polypeptide antibiotic derived from *Streptomyces* sp. It became approved by the US Food and Drug Agency (FDA) in 1964 for the treatment of gestational trophoblastic neoplasia, metastatic, non-seminomatous testicular cancer, and various pediatric cancers. Toxicity has, however, limited its use. ActD has also been widely used as a chemical probe in the study of rRNA synthesis and the nucleolar stress response. Robert Perry, already in 1962, published a study featuring a fundamental discovery: rRNA was made in the nucleolus [64]. ActD, when used at low concentrations (~30 nM), it suppresses the incorporation of radiolabeled nucleotides into nucleolar and cytoplasmic RNA, but at that concentration, it shows no major effect on the synthesis of tRNA, 5S rRNA, and nuclear RNA. Thus, this study introduced two key concepts for the field: the site of the rRNA synthesis and its first chemical inhibitor, ActD. Other studies soon followed, reporting nucleolar disintegration and the formation of nucleolar caps in cells upon treatment with low doses of ActD [65,66]. In 1970, Perry et al. quantified the varying sensitivities to ActD among the RNA species, suggesting a dose-dependent relationship of transcription inhibition [67]. Importantly, ActD inhibits RNA synthesis via its interaction with guanine residues on DNA, thereby inhibiting the activity of DNA-dependent RNA polymerases; Pol I showed the highest sensitivity, thought to reflect the GC-rich composition of ribosomal DNA.

ActD comprises two cyclic pentapeptide lactone rings and a heterocyclic planar aromatic ring system. The latter is mainly responsible for the DNA intercalation capacity of the compound, which is preferentially inserted between guanine-cytosine pairs, providing ActD with its GC-rich intercalation selectivity. Hydrogen bonding and hydrophobic interactions between the pentapeptide chain residues and the deoxyguanosine residues further stabilize the ActD-DNA interaction. As a result, the double helix cannot unwind, disrupting the activity of RNA polymerases and hence transcription.

As commonly observed with other intercalating agents, TOP1 (topoisomerase 1) and/or TOP2 (topoisomerase 2) inhibition could, at least in part, mediate the observed cytotoxicity. Trask et al. reported that treating nuclei with ActD stimulates the formation of covalent intermediates between TOP1 and DNA [68]. Based on their findings, the authors proposed an explanation for the high sensitivity of rRNA synthesis to ActD, as TOP1 appears concentrated in the nucleolus and is catalytically active on the rDNA. Further studies to elucidate the mechanism of rDNA transcription inhibition suggested that ActD interacts with G-Quadruplex (G4) DNA motifs found in oncogenic promoters, for example, c-MYC and telomeric repeats [69,70,71]. Note that ActD at higher concentrations, known to inhibit also Pol II activity, generates DNA double-strand breaks and the formation of γ-H2AX foci [72]. Exploring the cellular effects of the transcriptional blockade, p53 stabilization, upregulation of p21, and G_1_ arrest upon treatment were seen with 20 nM of ActD [73]. p53 stabilization had already been reported earlier by Kastan et al. at a concentration of only 0.45 nM [74], initially thought to be induced by the DNA damaging activity of ActD, which, however, occurs at higher concentrations. p53 stabilization at lower concentration is mediated by the increased binding of the 5S RNP complex with MDM2 [48]. Indeed, several papers indicated that ActD rapidly triggers the IRBC and p53 activation [48,49,75]. Altogether, these discoveries have turned ActD into the paradigm of Pol I inhibitors, even though the precise mechanism of inhibition is not fully understood. Interestingly, ActD was recently shown to specifically downregulate SOX2 expression in breast cancer and glioblastoma, SOX2 being a key regulator of stem cells’ self-renewal capacity, associated with glioblastoma aggressiveness and poor prognosis [76,77]. ActD reduced tumor growth in recurrent glioblastoma patient-derived models and increased overall survival [76]. It is quite extraordinary that almost 60 years after its approval by the FDA we are discovering new effects and potential application areas for this drug.

The intercalators aminacrine (also known as 9-aminoacridine, 9-AA) and ethacridine are anti-microbial agents used in disinfectant formulations. These are acridine derivatives, which are planar, aromatic DNA intercalators with a preference for GC-rich sequences [78,79]. Acridine derivatives, including 9-aminoacridine, amsacrine, and quinacrine, were found to induce p53 transcriptional activity and stabilize p53 protein by blocking its ubiquitination [80]. Aminacrine and ethacridine activate p53 in a DNA damage-independent manner, also triggering dose-dependent degradation of the catalytic subunit of Pol I, POLR1A (RPA194), and the IRBC [81]. Both compounds inhibited cell growth within the low micromolar range. A recent study revealed that aminacrine interferes with both rRNA synthesis and rRNA processing while also having the ability to bind RNA *in vitro*, which may play a role in the pre-rRNA processing alterations observed [82]. Along the same line, quinacrine, used in malaria prevention and treatment [79,83], was identified in a drug repurposing screen conducted in leukemia cells. Bioinformatic analyses of gene enrichment and drug correlations revealed mechanistic signatures related to RiBi and a strong drug-drug correlation to a known investigational Pol I inhibitor, ellipticine [84]. In ovarian cancer cells, quinacrine downregulated the expression of nucleostemin and POLR1A, triggering nucleolar stress [85]. Several compounds in the acridine family may act as TOP1 and/or TOP2 inhibitors, but the mechanisms involved remain poorly understood [78].

4-aminoquinolines contain planar aromatic moieties with GC-rich selective DNA intercalation capacity. A study published by our group showed that amodiaquine, an FDA-approved drug used against malaria, inhibited transcription of rDNA and enhanced proteasomal degradation of POLR1A followed by p53 stabilization [86]. In agreement, earlier studies had indicated stabilization of p53 in amodiaquine-treated cells [87,88]. We could also show that amodiaquine inhibited the proliferation of several colon cancer cell lines [86]. While amodiaquine is a well-known autophagy inhibitor, our findings support a second, additional mechanism related to RiBi inhibition.

The intercalating anthracyclines doxorubicin and mitoxantrone display broad antitumor activity against several types of human cancers and inhibit rDNA transcription [34]. Doxorubicin appears to be the most potent member of this class and is effective against solid tumors. Due to their structural characteristics, these compounds are powerful DNA intercalators; but besides the formation of DNA adducts, they induce oxidative stress, cause DNA damage, and they are TOP2 poisons [89,90]. It is important to keep in mind that doxorubicin has the ability to trigger histone eviction from chromatin [91]. Doxorubicin and mitoxantrone inhibit rRNA synthesis and induce nucleolar disruption; however, the mechanism of inhibition is not understood in detail. TOP2A is involved in RNA pol I PIC formation and transcription [92], suggesting that interference with TOP2A by intercalating agents may play a role in the overall negative effect on rDNA transcription.

### 3.2. DNA Alkylating Agents

In the group of alkylating agents, two of the most widely used in cancer chemotherapy, the platinum-based compounds cisplatin and oxaliplatin, target the nucleolus and interfere with rRNA synthesis [93,94]. Cisplatin represents a cornerstone of cancer chemotherapy and is used against more than 18 cancer types. It displays high efficiency but is also toxic for certain normal cells, which motivated the development of analogs such as carboplatin and oxaliplatin. Platinum compounds interact electrostatically with DNA; the electrostatic interaction is followed by complexation with the N-7 atoms of adenine or guanine, leading to intrastrand cross-linking. As a result, the DNA tertiary structure is disrupted, and the complexion site unwinds. Consequently, the high-mobility group (HMG) domain proteins bind the DNA damage site, preventing DNA replication and inducing cell death. HMG proteins include UBF, which is then inhibited from promoting Pol I transcription [95,96,97]. Cisplatin also impairs rDNA transcription by the re-distribution of PIC components to the outer part of the nucleolus [93]. Interestingly, oxaliplatin was considered to have a different or additional molecular target from cisplatin or carboplatin. Oxaliplatin creates fewer cross-links per base than cisplatin and has been shown to exert its cytotoxic effect even on tumor cell lines resistant to cisplatin and carboplatin, while alterations conferring resistance to cisplatin were not found to induce resistance to oxaliplatin [94]. Oxaliplatin is used as a first-line treatment of colorectal cancer in the FOLFOX (folinic acid, fluorouracil, oxaliplatin) regimen, while it has a different side-effect profile than its sister compounds. These mechanistic and clinical differences might, in part, be explained through oxaliplatin-induced RiBi stress rather than DNA damage [94]. A comparison of oxaliplatin and cisplatin side by side revealed that oxaliplatin induces a more rapid re-localization of nucleolar components [98]. Examples of clinically approved drugs that interfere with RiBi are listed in Table 1. One conventional approach for RiBi-targeted drug discovery is studying the structure-activity relationship and the subsequent optimization of pharmacophores known to interact with the RiBi machinery. To this end, Sutton and co-workers recently studied several platinum-based, oxaliplatin-like compounds and their effect on the nucleolus by quantifying NPM1 translocation [99]. The results defined a set of structural constraints for Pt(II) compounds to induce the IRBC and revealed two additional analogs with robust nucleolar stress-inducing capacity, named DACH-platin and Benzaplatin.

Mitomycin C is a natural antibiotic that, after chemical or enzymatic reduction, is transformed into a reactive metabolite with powerful alkylating activity. It cross-links DNA preferentially at G-rich sequences and induces DNA damage [100]. Mitomycin C is thought to inhibit rDNA transcription by causing cross-links in the GC-rich rDNA and thus interfering with the Pol I transcription machinery [34,101]. A related compound, streptonigrin, appeared as an interesting hit in a screening for RiBi interfering agents [102]. It is a natural antibiotic and a member of the group of agents that possess the aminoquinone moiety, e.g., mitomycin C. Streptonigrin is genotoxic and inhibits RNA synthesis, causes DNA strand breaks, induces the formation of DNA adducts, and inhibits TOP2 [103]; it was tested in chemotherapy but was discontinued due to toxicity and is unlikely to be repurposed.

**Table 1:** Examples of drugs approved for clinical use that impair ribosome biogenesis.

Compound	Mechanism	RiBi Target	Reference
Actinomycin D	DNA Intercalator	rRNA synthesis	[67,68]
Mitoxantrone	DNA Damage, TOP2 inhibitor.	rRNA synthesis	[34,89]
Doxorubicin	DNA Intercalator, TOP2 inhibitor	rRNA synthesis	[34,90,104]
Oxaliplatin	DNA Cross-linker	rRNA synthesis, processing	[94,99,105]
Cisplatin	DNA Cross-linker	rRNA synthesis	[93,95,96]
Carboplatin	DNA Cross-linker	rRNA synthesis	[94]
Mitomycin C	DNA Alkylator, TOP2 inhibitor	rRNA synthesis	[106,107,108]
5-Fluorouracil	Antimetabolite	rRNA processing	[34,109,110]
Methotrexate	Antimetabolite	rRNA synthesis	[111,112]
Camptothecin	TOP1 Inhibitor	rRNA synthesis	[113,114]
Etoposide	TOP2 Inhibitor	rRNA processing	[115,116]
Aminoacridine	DNA Intercalator	rRNA synthesis	[81]
Ethacridine	DNA Intercalator	rRNA synthesis, processing	[81]
Amodiaquine	Several + Autophagy Inhibitor	rRNA synthesis	[86]
Rapamycin	mTOR Inhibitor	rRNA synthesis	[31,117]
Mycophenolic acid	IMPDH2 Inhibitor	rRNA synthesis	[118]

### 3.3. Antimetabolites

Antimetabolite drugs have been extensively used in cancer chemotherapy; examples include methotrexate and 5-fluorouracil (5-FU). Methotrexate is a classical antifolate used for the treatment of several cancers, while at the same time, it is the most widely prescribed disease-modifying antirheumatic agent used to treat psoriasis or rheumatoid arthritis [112]. It is an analog of folic acid and inhibits the enzymatic activity of dihydrofolate reductase. Folate has an essential role in the synthesis of thymidylate and purine bases, and its deficiency caused by dihydrofolate reductase inhibition results in cell death. Importantly, methotrexate treatment of cells impairs Pol I transcription and decreases nucleolar size [34].

5-FU is an analog of uracil used in the treatment of breast, head and neck, and colorectal cancer, among others. It received FDA approval in 1962, two years before ActD. It is a prodrug that in cells is activated to 5-fluoro-dUMP (FdUMP) and 5-fluorodUTP (FdUTP). FdUMP inhibits the enzymatic activity of thymidylate synthase, an enzyme that catalyzes dUMP to dTMP conversion, depleting the intracellular deoxynucleotide pool; hence, it suppresses DNA synthesis and repair, causing DNA damage [110,119,120]. Not only that, FdUTP is incorporated into RNA, particularly rRNA, and inhibits rRNA processing by interfering with the maturation of pre-RNA. Treatment with 5-FU activates IRBC and p53 [109]. The 5-FU derivative carmofur was shown to block rRNA processing in a screen conducted in yeast cells [102].

Recent studies have provided additional insights into the 5-FU mechanism of action. Using a proteome-wide cellular thermal shift assay to analyze drug-protein interactions, it was confirmed that 5-FU affects not only rRNA but several other categories of RNA species [121]. 5-FU is incorporated into rRNA and subsequently into functional ribosomes, resulting in altered translation [122]. As mentioned, 5-FU together with oxaliplatin are included in the FOLFOX standard treatment for colorectal cancer. It is possible that the effectiveness of FOLFOX stems from a combination of RiBi inhibition, altered protein synthesis, and accumulation of DNA damage in the cancer cells.

### 3.4. Plant-Derived Alkaloids

Camptothecin is a natural alkaloid, and its derivatives (topotecan and irinotecan) represent the first class of type I TOP inhibitors used in the clinic. TOP1 is associated with rDNA transcription different from TOP2A; inhibition of the enzyme leads to the formation of TOP1-DNA adducts, inhibiting transcription and inducing DNA damage. Camptothecin has been shown to interrupt rRNA synthesis and early rRNA processing while in parallel inducing the formation of nucleolar caps [34,113]. Interestingly, a recent in vitro study suggests that camptothecin disrupts Pol I transcription through DNA intercalation *per se*, independently of TOP1 inhibition [123]. Topotecan also inhibits rDNA transcription and intracellular relocalization of TOP1 [124,125]. Etoposide is a semi-synthetic derivative of 4-epipodophyllotoxin, a plant-derived alkaloid. It is a TOP2 inhibitor, capable of inducing DNA damage, and its effect depends on the cell cycle phase, with optimal efficacy during the S and G2 phases. Etoposide disrupts late processing of rRNA and, similarly to camptothecin, induces the formation of nucleolar caps [115].

### 3.5. Non-Intercalating Antibiotics

Rapamycin, a natural macrolidic antibiotic, was initially used for its immunosuppressive properties, e.g., blocking T-cell activation. It is a well-known inhibitor of the mTOR pathway, blocking the activation of the serine/threonine kinase S6K1. Since the PI3K-AKT-mTOR signalling pathway is frequently upregulated in cancer, rapamycin raised expectations as an anti-cancer therapeutic agent. While pharmacological properties limited its application in cancer treatment, the effects of rapamycin inspired the development of numerous more potent derivatives, some of which obtained FDA approval for certain cancers. The inhibition of the mTORC1 complex by rapamycin downregulates RiBi. Among several effects, rapamycin inactivates an essential component of Pol I transcription machinery, TIF-1A, by altering its phosphorylation and inducing its accumulation in the nucleoplasm [31,126,127,128].

Mycophenolic acid was originally used as an immunosuppressant but is also reported to inhibit rRNA synthesis, disrupt the nucleolus, trigger IRBC and activate p53 [118]. This effect may in part stem from the mycophenolic acid mediated inhibition of IMP dehydrogenase-2 (IMPDH2), a rate-limiting enzyme for *de novo* guanine nucleotide biosynthesis [129]. IMPDH2 was found to be overexpressed in high-grade gliomas, and inhibition of IMPDH2 activated IRBC, resulting in glioma cell growth arrest [129]. Functionally, IMPDH2 maintains elevated rDNA transcription, as Pol I relies on the IMPDH2-dependent guanine nucleotide biosynthesis, while the normal glial cells sustain Pol I transcription by using the salvage pathway, suggesting a therapeutic selectivity window [129].

### 3.6. Other Compound Classes That May Affect Ribosome Biogenesis

**Figure 1:**
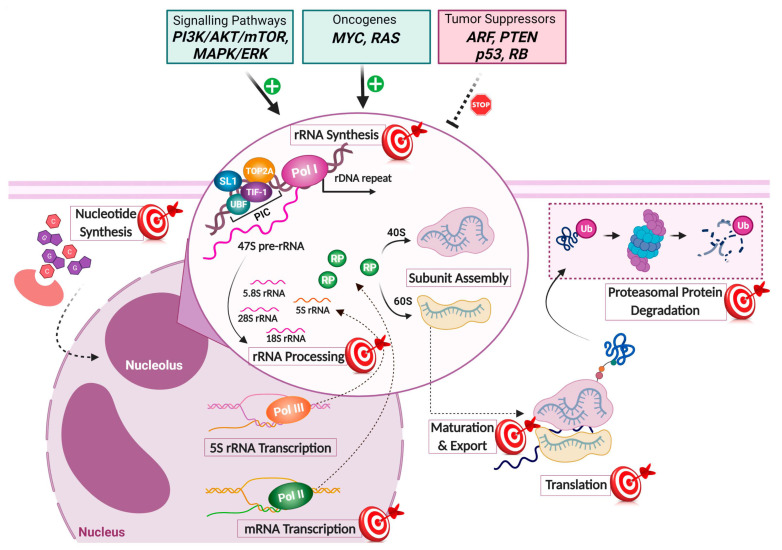
Cellular targets and processes for small molecules that directly, or indirectly, can interfere with ribosome biogenesis. Figure was created with Biorender.com under academic license.

As mentioned, RiBi is a complex process that, in addition to Pol I, requires Pol II and III and intact RNA processing types of machinery, such as splicing and regulated turnover. A genome-wide RNAi screen revealed how a number of proteins in the small subunit processosome, the ubiquitin-proteasome system, and the splicing apparatus are required to support ribosomal 40S subunit biogenesis [130]. Similarly, a genome-wide RNAi screen for processes supporting 60S subunit biogenesis indicated the importance of transcription, splicing, translation, protein degradation and the polyamine synthesis pathway [131]. Taking these and other studies into account, it is not surprising to see that drugs from several compound classes have a negative impact on RiBi, often in an indirect manner through poorly understood mechanisms. This is illustrated in Figure 1. An example among such compounds is the proteasome inhibitor bortezomib, which interferes with late rRNA processing [34]. Bortezomib, FDA-approved for use in lymphoma and multiple myeloma, also triggers changes in the nucleolar structure [132]. It is well established that proteasome inhibitors cause nucleolar aggregations of proteins and RNA [133,134]. As another example of dual-effect drugs, the translation inhibitor homoharringtonine was shown to affect late rRNA processing [34]. It is a cephalotoxine ester discovered in 1963, and today is known as omacetaxine mepesuccinate (Synribo), and it is used in the treatment of chronic myeloid leukemia in some countries [135,136]. Furthermore, some adenosine analogs and cyclin-dependent kinase (CDK) inhibitors impair Pol II transcription and RiBi, yet these compounds remain in development and clinical testing and they will be discussed in Section 4.7.

## 4. Drug Discovery: Identification and Development of molecules That Inhibit Ribosome Biogenesis

**Figure 2:**
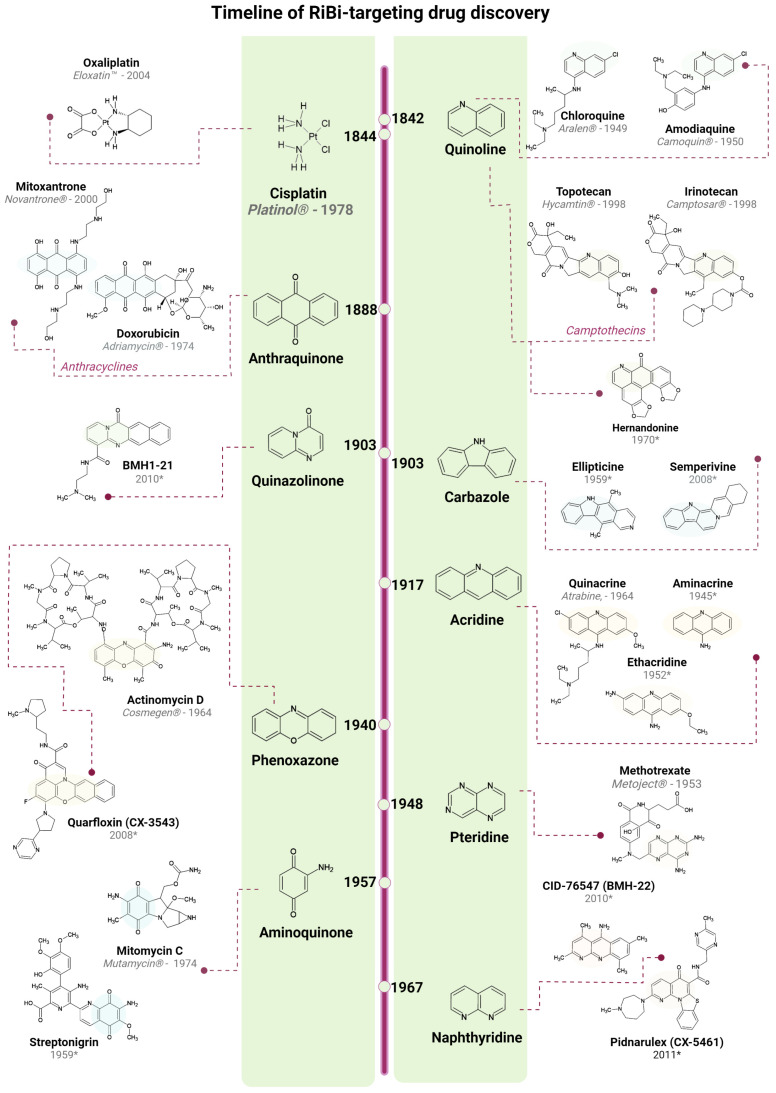
A schematic timeline of the most common substructures found in RiBi inhibitory cancer drugs. Drugs known to interact with the RiBi machinery are listed along with their name, trade name, and clinical approval year. Investigational drugs are shown with their name and the year they first appeared in the literature in italics with an asterisk. The drugs are grouped based on their substructure, shown on the green-colored box, with the initial discovery or synthesis year. The figure was created with Biorender.com under academic license.

Given that several oncogenic pathways and biosynthetic processes are linked to RiBi, and that the mechanisms of many chemotherapeutic drugs are closely connected to diverse RiBi steps, the interest in obtaining Pol I specific inhibition in cancer increased. This led to the discovery of several new compounds, some with promising anti-cancer activity, which are discussed in the following section. This is also summarized in a timeline in Figure 2. Novel tools for drug screening, methods for high-content image-based microscopy, in-silico drug modelling, and genetic tools have advanced our knowledge about the nucleolus and the prospects for drug discovery. Common themes are emerging among the various screening strategies and assays that aim to find RiBi modulating compounds. A few published studies have used ribosomal proteins (RP) fused with a fluorophore e.g., GFP, as a readout [102]. The RP-GFP can be used to analyze the possible entrapment of pre-ribosomal particles in the nucleoplasm, or the nucleolus, assuming that the RP-GFP fusion is stably incorporated into ribosomes. There are other tags than GFP; for instance, the HaloTag epitope (based on Promega’s HaloTag technology) [137]. This phenotypic approach can lead to the identification of many compounds since the RiBi process is sensitive, while secondary validation screens are needed to identify the most promising hits. Several compounds that affect the nucleolus may do so indirectly through inhibition of transcription, translation, or blockade of intracellular transport. This must be considered when designing the assays, as it may affect reporter construct expression or other aspects of the screen.

**Figure 3:**
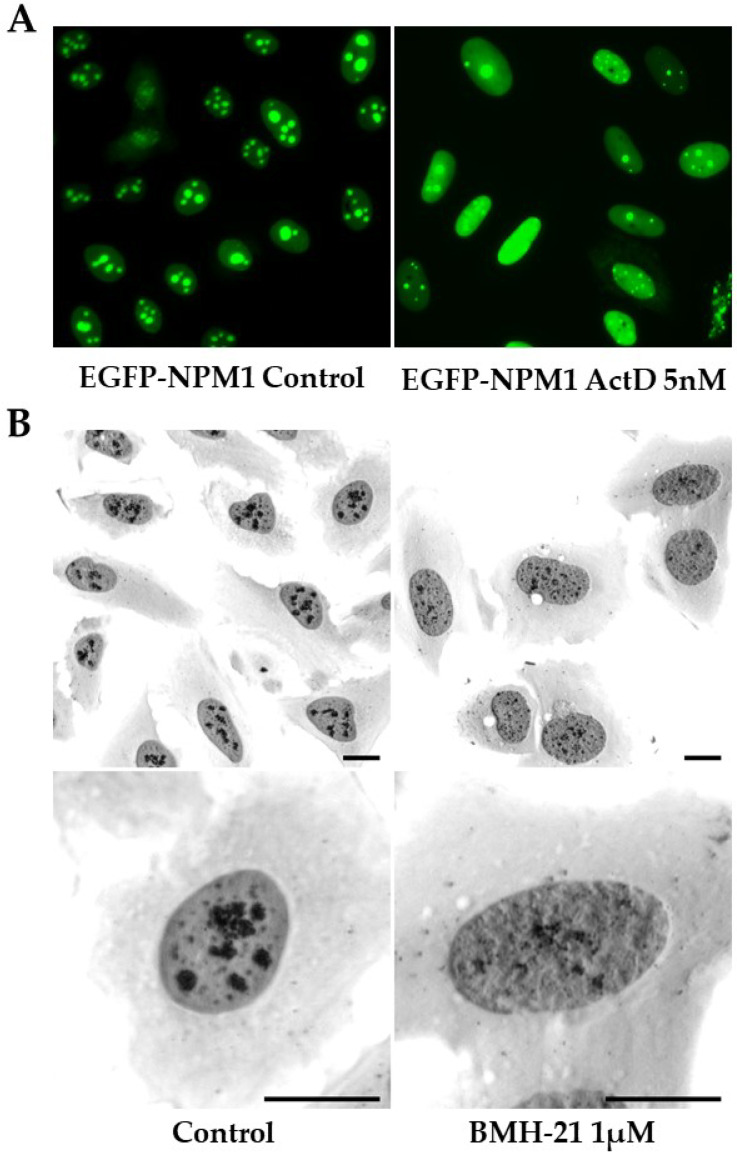
Examples of how ActD and BMH-21 affects nucleolar markers. (**A**) EGFP-NPM1 intracellular re-distribution upon treatment with a low concentration of ActD (5nM). On the left control, live unfixed U2OS cells; on the right, U2OS cells treated with ActD. The solvent for ActD in this experiment was ethanol. Note the more intense nucleoplasmic signal, while the round nucleolar areas have shrunken in the ActD-treated sample. Image by M. Lindström. (**B**) Nucleolar disruption induced by BMH-21 in U2OS cells. AgNOR staining of U2OS cells treated with DMSO (left) or 1 μM of BMH-21 (right) for six hours. Zoom-in of select cells in the lower row. Scale bar 10 μm. Image by A. Zisi.

From a historical point of view, one may consider that the first pilot screens of compounds in cells were conducted using immunostaining for the major nucleolar protein NPM1 (also known as B23 or nucleophosmin). A shift in NPM1 location from the nucleolus to the nucleoplasm was used as a readout when testing various cytostatics. This was known as the “B23 translocation assay” and thought to reflect a cessation of rDNA transcription. Later, the pattern of NPM1-GFP was used as a readout in living cells (see e.g., Figure 3A. Among other drugs, NPM1 translocation was seen in cells exposed to ActD, camptothecin, toyocamycin, and doxorubicin [104,108,111,138,139]. Today, it is established that many nucleolar antigens translocate out of the nucleolus, or display other types of staining patterns, following inhibition of rDNA transcription. Indeed, the nucleolus can be viewed as a multiphase liquid droplet, and RiBi inhibitors may affect fundamental biophysical properties of the nucleolus including phase separation [27,140,141]. For example, nucleolar fibrillar centers segregate to the nucleolar periphery, where they merge to form caps in response to Pol I inhibition [141].

### 4.1. Quarfloxin, CX-3543

CX-3543 (quarfloxin) is a fluoroquinolone and phenoxazole derivative that disrupts the interaction of nucleolin with G-quadruplex structures in rDNA resulting in transcription inhibition [142]. CX-3543 curbs transcription elongation by hindering this interaction, resulting in reduced levels of 47S rRNA and subsequently inducing p53, leading to cell cycle arrest and apoptosis. CX-3543 underwent clinical trials, completing phase I and advancing into phase II for the treatment of low to intermediate grade neuroendocrine and carcinoid tumors (NCT00780663); however, due to excessive albumin binding, it was discontinued [7]. CX-3543 was reported to induce the formation of γ-H2AX foci and stabilize G4s formed on oncogenic promoters such as c-MYC, c-KIT, and telomeric ends in vitro [143]. Such stabilized G4 foci, demonstrated by immunofluorescence, were colocalized with 53BP1 DNA damage foci in cells treated with CX-3543.

### 4.2. Pidnarulex, CX-5461

CX-5461 (pidnarulex), a naphthyridine derivative, was identified in a high throughput drug screening campaign for selective Pol I inhibitors. It exhibited improved specificity and efficacy for Pol I transcription inhibition at low concentrations, with IC_50_s below 100 nM and significantly higher inhibitory activity towards Pol I, compared to Pol II [144,145]. CX-5461 inhibits the interaction between SL1/TIF-IB and the rDNA promoter, preventing the assembly of Pol I machinery. The drug activates p53 and induces apoptosis. In p53-null cancer cell lines, it activates p53-independent G2 cell cycle arrest mediated by ATM/ATR signaling [146]. CX-5461 has been tested in vitro in a wide range of cell lines, showing promising efficiency as a monotherapy or as part of combinatorial treatments. For example, it elicited anti-cancer effects in preclinical models of lymphomas, leukemia, neuroblastoma, prostate, breast, small cell lung, and ovarian cancer [147,148,149,150,151]. A derivative of CX-5461, denoted RAM-589.555, has also been described; it suppresses RiBi and ameliorates experimental autoimmune encephalomyelitis with implications for multiple sclerosis [152]. CX-5461 advanced into clinical trials for hematological malignancies, completing a phase I dose-escalation study with promising clinical results, including for solid tumors; it is currently in phase I/II trials. Several studies following up on the development of CX-5461 provided novel mechanistic insights, suggesting a multi-target drug. CX-5461 is a G4 stabilizer that induces replication fork blockade and single-strand DNA breaks [48,49,50,51,52]. These effects were observed at concentrations approximately equal to the IC_50_s of CX-5461. BRCA-deficient cell lines may be particularly susceptible to the G4-mediated DNA damage, which requires the BRCA and non-homologous end-joining pathways to be resolved. A phase I clinical trial for patients with BRCA1/2 deficient tumors was initiated (NCT02719977), showing good tolerability and preliminary efficacy in patients with homologous recombination deficient tumors.

Bruno et al. reported that the primary cytotoxic mechanism of CX-5461 involves TOP2 poisoning and went on to demonstrate that previously sensitive CX-5461-resistant lymphoma cells were collaterally resistant to doxorubicin [153]. Importantly, TOP2A knockdown in mouse and human cancer cell lines causes resistance to doxorubicin and CX-5461, but not to ActD [153]. TOP2A was identified as a target for CX-5461 in an independent study [154]. In contrast, while CX-5461 appears synergistically effective in combination with TOP1 inhibitors in the treatment of neuroblastoma, Pan et al. pinpointed TOP2B, rather than TOP2A, as a target of CX-5461 [155]. Inhibition of TOPs and Pol I is clearly a potent growth inhibitory mechanism, but the inhibition of TOP2B is of some concern due to possible toxic side effects. Inhibition of TOP activity explains at least some of the DNA damage seen in cells treated with CX-5461. A new compound under development, different from CX-5461 and inhibiting Pol I, is denoted PMR-116 (see ref. [7]) and has a different profile. DNA damage in combination with Pol I inhibition is not necessarily a disadvantage in cancer therapy, but it makes the interpretation of cellular phenotypes more challenging.

### 4.3. BMH-21 and CID-765471

BMH-21 is an acridine-like quinazolinone derivative and a DNA intercalator with GC-rich sequence selectivity. It was initially discovered in a cell-based high-content screen for compounds activating p53, whereas it showed no DNA damage-inducing activity [128]. Later it was described in more detail how BMH-21 inhibits rRNA transcription by impairing transcription elongation by Pol I, and triggering proteasomal degradation of POLR1A [156,157]. A recent study further corroborated these findings by employing an in vitro transcription assay and the in vivo native elongating transcript sequencing in yeast, showing that BMH-21 inhibits initiation, clearing of the promoter, and elongation by Pol I [158]. BMH-21 evokes p53 activation and induces nucleolar cap formations, but in contrast to CX-5461, it does not induce γ-H2AX foci. BMH-21 exhibited solid anti-cancer efficacy across the NCI60 panel of cancer cell lines, and BMH-21 repressed tumor growth in mice [157]. The cytotoxic response to BMH-21 only partially depends on p53 [157], and depletion of RPL11 or RPL5 did only partially prevent p53 stabilization in U2OS cancer cells exposed to BMH-21 [86]. Figure 3B illustrates how the treatment of U2OS cells with BMH-21 causes a rapid dispersal and shrinkage of AgNOR positive nucleolar regions.

The main mechanism of action is considered to be mediated by the intercalation of BMH-21 between the GC-rich sequences of rDNA, inhibiting transcription elongation and initiation. The actual trigger of POLR1A degradation is not fully understood. The BMH-21 derivatives BMH-9, -22, and -23 were also reported to inhibit rRNA synthesis, induce nucleolar stress, and POLR1A degradation [126,127]. BMH-21 was the first inhibitor identified to directly affect the Pol I machinery by triggering the degradation of one of its subunit components. As an intercalator, effects outside the nucleolus are to be expected at higher concentrations. BMH-21 was implicated in G4 stabilization by studies that showed its interaction with G4 structures of the c-KIT and c-MYC promoters, leading to the downregulation of c-MYC [159]. On the contrary, Xu et al. showed no effect in G4 stabilization by using two different in vitro methods [143]. This was also supported by a more recent independent study [160].

Using a cell-based phenotypic screen for compounds that could disrupt the nucleolus (U2OS cancer cells expressing GFP-RPL37), a compound denoted CID-765471, and identical to BMH-22, was identified [81]. CID-765471 is similar to aminacrine, ethacridine, and BMH-21 in that they share the ability to induce POLR1A degradation and stabilize p53 in the absence of DNA damage.

Regardless of the remarkable in vitro efficacy of the emerging POLR1A-degrading molecules, no compound has progressed into clinical testing to date. A possible explanation could be structural limitations. For instance, BMH-21 inhibits the human Ether-a-go-go related gene (hERG), a predictor of QT prolongation and possibly fatal cardiac arrhythmia. The authors performed a structure-activity relationship study among a series of pyridoquinazolinonecarboxamide analogs to optimize the structural and pharmacokinetic features of BMH-21, resulting in the generation of new scaffolds with decreased off-target activity while retaining the desired POLR1A degradation effect [161].

### 4.4. Alkaloids and Lactones with RiBi Inhibiting Activity

A family of planar alkaloids named ellipticines is another class of DNA intercalators, displaying a strong preference for GC-rich sequences. Ellipticine and 9-hydroxyellipticine (9-OH) have been reported to inhibit rRNA synthesis with high selectivity and efficiency [162]. The inhibition is ATM/ATR- and TOP2-independent; mainly, 9-OH disturbs the formation of PIC by targeting the binding of SL1 to the rDNA promoter. However, several studies indicate alternative or additional mechanisms; it also seems to exert anti-TOP1/2 activity and induce Reactive Oxygen Species (ROS). Several clinical trials have evaluated ellipticine derivatives, but adverse side effects hampered further development.

With a mechanism reminiscent of BMH-21, hernandonine, a natural, planar polycyclic alkaloid, was reported to inhibit rRNA synthesis and induce nucleolar stress and POLR1A degradation [163]. It is effective at low micromolar levels while inducing cellular apoptosis in solid tumor cell lines. Sempervirine, an alkaloid of *Gelsemium sempervirens* and putative inhibitor of MDM2, was also found to cause nucleolar stress and degradation of POLR1A with an overall decrease in rRNA synthesis. Interestingly, notable anti-proliferative effects of the compound were seen in p53 null cells coupled to a decrease in E2F-1 levels and without inducing DNA damage [164].

Triptolide is a natural lactone that potently inhibits rRNA synthesis, triggering immediate nucleolar segregation and activation of the p53 pathway. In vivo data using A549 xenografts revealed effective tumor size reduction [165]. Interestingly, accumulating evidence suggests that triptolide is a pan-RNA polymerase inhibitor. It interacts with components of Pol II machinery and induces rapid degradation of the catalytic subunit of Pol II, RBP1 [166,167,168]. Moreover, it interrupts the TFIIIB association at tRNAs and 5S rRNA promoters, downregulating Pol III [169].

### 4.5. Metarrestin

Metarrestin, a synthetic compound, was discovered in a high-throughput drug screen for compounds targeting a metastatic cancer cell line, using the perinucleolar compartment as a phenotypic marker of metastatic potential [170]. The perinucleolar compartment is a dynamic subnuclear body located in the periphery of the nucleolus, highly enriched in non-coding RNAs and RNA-binding proteins. It may function in RNA metabolism and in the Pol III machinery [171]. Cancer cells may have the perinucleolar compartment, but it is usually not seen in normal cells. Its prevalence has been positively correlated with metastatic potential, disease progression, and poor overall survival in breast, colorectal, and ovarian cancer [171].

Following lead optimization, metarrestin was found to disrupt the nucleolar structure in a fashion similar to ActD and indirectly inhibit Pol I transcription; the mechanism may involve interaction with the translation elongation factor eEF1A2 [170]. Metarrestin effects were found to be DNA damage-independent, while, unlike other rRNA synthesis inhibitors, it did not induce apoptosis regardless of the p53-status. Moreover, the drug evoked a disassembly of the perinucleolar compartment and inhibited cancer cell invasion. While the drug only modestly affected primary pancreatic tumor growth, metastatic progression was stalled in different mouse models of human cancers, and improved survival in a metastatic pancreatic cancer xenograft model was seen [170]. Metarrestin has now entered phase 1 clinical testing in patients with metastatic solid tumors (NCT04222413). Overall, this study was the first comprehensive attempt to connect RiBi inhibition to cancer invasion and metastasis, indicating a novel targeting approach using a candidate with favorable pharmacokinetic properties. In support, functional connections have been reported between RiBi and Epithelial-to-Mesenchymal Transition (EMT), a cellular program associated with cancer progression and metastasis, where epithelial cells acquire migratory and invasive properties [172]. EMT was reported to be sustained by the upregulation of RiBi during G1/S cell cycle arrest, as shown by the increased expression of PIC components and the enhanced association of Pol I, UBF, and Snail1, a transcription factor that promotes EMT, with the rDNA promoter. Inhibition of Pol I downregulated levels of pro-invasive mesenchymal proteins and curtailed cellular invasiveness. The connection of RiBi to EMT is further reviewed by Elhamamsy et al. [173].

### 4.6. Additional RiBi Targeting Compounds

Several other small molecules or peptides that inhibit RiBi have been described. Using a HaloTag selective labeling strategy, a malignant melanoma cell line was used to screen for compounds able to reduce ribosome content, and 5786 compounds were identified [137]. Following a secondary screen that relied on measurements of pre-rRNA, two compounds denoted Ribosome Biogenesis Inhibitors 1 and 2 (RBI1 and RBI2) were described. Some studies have utilized rDNA promoter-based constructs. For example, a yeast cell line with a stably integrated human Pol I promoter and rDNA was used, leading to the identification of cerivastatin sodium (a statin compound and HMG-CoA reductase inhibitor that is removed from market)**.** This statin compound demonstrated anti-proliferative effects in A2780 and H460 cancer cell lines [174]. A 22-amino-acid peptide was shown to disrupt binding between Pol I-associated factor Rrn3 (TIF-1A) and the Pol I complex subunit, causing nucleolar stress and cell death [175]. We envisage several novel small molecules being developed and tested over the coming years.

### 4.7. Targeting Other Cellular Processes Impacting on RiBi

As mentioned, RiBi relies on several other cellular functions, including nucleotide metabolism, Pol II and III transcription, splicing, nuclear import/export, protein synthesis, and degradation. The RiBi interfering activity of small molecules blocking these processes may at least to some extent contribute to their overall anti-cancer effect. Among them, we find inhibitors of CDK7, CDK9, c-MYC, mTOR pathway, mRNA splicing machinery, and molecules disrupting metabolic pathways. Below we briefly discuss a few promising candidates.

The development of Pol II transcription inhibitors that preferentially target malignant cells has been difficult. Targeting the transcription of mRNA affects multiple pathways, and consequently there are often side effects arising. Recent studies underscore the importance of Pol II transcription of Alu-repeats in order to maintain normal nucleolar structure and function [176], and the role of Pol II activity around nucleoli supporting transcription of rDNA by Pol I to drive RiBi [177]. A compound that has been used in experimental research for decades is the adenosine analog DRB (5,6-dichloro-1-b-D-ribofuranosyl-benzimidazole), a Pol II transcription blocker [153]. Exposure of cells to DRB causes the fibrillar components of the nucleolus to unravel into necklace-like structures, most likely representing extended linear arrays of rDNA being transcribed [178]. DRB interferes with rRNA processing; it is an inhibitor of CDK9 and casein kinase 1 and 2, specifically affecting the elongation step. Among novel compounds with promising clinical effects are CDK inhibitors, several of which alter nucleolar structure, activate p53, and disrupt rRNA processing [53,179,180]. CDK1, CDK2, CDK7 and/or CDK9 inhibitors affecting rRNA processing include, for example roscovitine (seliciclib), olomoucine, and flavopiridol (alvocidib) [34,181]. A detailed understanding of how these compounds disrupt rRNA processing and the nucleolar structure is missing but may in addition to the above mentioned events involve downregulated production of U8 small nucleolar RNA by Pol II [181]. Clearly, much more is to be discovered about the intricate relations among the RNA polymerases and RiBi.

Inhibitors of mRNA processing and turnover may negatively impact proteins involved in RiBi through various mechanisms. Recently, small-molecule inhibitors of EIF4A3, a core RNA binding helicase in the exon junction complex, and with a second function in the rRNA processing machinery, were shown to markedly impair RiBi, activate p53, and result in nucleolar shrinkage [182]. Inhibitors of Pol II, splicing, and the exon-junction complex may not only activate p53 through RiBi stress and IRBC, but also by altering MDM2 splicing, or reducing MDM2 transcription, thereby allowing p53 to escape proper control by MDM2 [55,182].

The interplay between RiBi and DNA replication is an emerging area of research [183]. The critical role of cellular ATP and GTP pools in nucleolar rRNA synthesis was noted long ago [184]. Limiting nucleotide availability by blocking *de novo* purine and pyrimidine synthesis pathways to impair RiBi is emerging as a promising strategy, with interesting experimental data from glioblastoma models [185]. As an example, besides IMPDH2 (previously discussed), impaired de novo biogenesis of pyrimidines through inhibition of DHODH (dihydroorotate dehydrogenase) downregulates rRNA synthesis and triggers IRBC in glioma cells [186]. In another study, DHODH inhibitors were shown to trigger IRBC, p53 activation, and replication stress in cancer cells [187]. It is interesting that DHODH inhibitors are frequently found in screens for compounds that activate p53. Besides RiBi and replication stress feeding into p53, some inhibitors may increase the synthesis of p53 protein [55,188].

An area of new discoveries concerns the less well-understood role of glutamine in RiBi. It is known that cancer cells are critically dependent on glutamine (“glutamine addiction”) due to its involvement in many metabolic processes [189]. Upon depletion of the glutamine synthetase GLUL, a metabolic enzyme, a specific 40S biogenesis defect appeared in HeLa cells. The normal function of GLUL is to catalyze L-glutamine production by adding ammonia to glutamate. However, nucleotides were not limiting for rRNA synthesis in cells depleted of the enzyme GLUL, but rather loss of GLUL led to errors in rRNA processing [130]. The compound acivicin was found to inhibit RiBi in yeast cells [102]. Acivicin is an analog of glutamine and inhibits gamma-glutamyl transferase, interfering with glutamate metabolism. Acivicin was studied previously as an anti-cancer agent, but clinical trials were not successful due to excess toxicity. It is plausible that acivicin may affect RiBi due to its impact on glutamine metabolism or enzymes in this pathway. The role of intracellular glutamine synthesis in cancer cell RiBi warrants further investigations.

### 4.8. Inhibitors of RiBi in Yeast

The family of AAA-ATPases (AAA—*ATPases* Associated with diverse cellular Activities) has emerged as a potentially important and druggable protein class. For example, compounds that inhibit the AAA-ATPase p97 (a k a VCP/Cdc48) and distort proteostasis were developed as potential anti-cancer drugs or for the treatment of neurodegenerative diseases. A metabolite of the anti-alcohol abuse drug disulfiram was found to target cancer cells through Npl4, an essential cofactor of p97 [190].

In yeast cells, two inhibitors that specifically target the maturation of ribosomal subunits rather than acting as inhibitors of mRNA translation have been identified. Interestingly, both inhibitors target AAA-proteins. The first one, diazaborine, blocks the large subunit formation in yeast by blocking the hexameric AAA-ATPase Drg1, mechanistically related to p97 [191]. Drg1 induces the ATP-dependent release of pre-60S shuttling and maturation factor Rlp24. Thus, Rlp24 release is inhibited by the drug diazaborine that prevents ATP hydrolysis and blocks the recycling of Drg1. This prevents additional ribosome subunit export since Rlp24 is not properly shuttling in the cell. The second inhibitor, Rbin1, acts as an inhibitor of the Dynein-like AAA-ATPase Mdn1 (midasin) [192]. Mdn1 has a role in assembling nucleolar precursors of the 60S subunit. The ribozinoindoles (“Rbins”), are potent and reversible triazinoindole-based inhibitors of RiBi in yeast cells. Conceptually, this mode of RiBi inhibition remains to be further explored and tested in mammalian cells.

Haemanthamine is an alkaloid from the *Amaryllidaceae* plant and has been studied as a novel anti-cancer agent. Haemanthamine binds to the *Saccharomyces cerevisiae* 80S ribosome, targeting the A-site cleft on the large ribosomal subunit, thereby altering rRNA positioning to block translation elongation. Interestingly, haemanthamine inhibits RiBi and activates the IRBC in human cancer cells [193]. There is currently an interest in compounds that affect the mature ribosome function in human cells; for reviews, see [194,195,196].

### 4.9. Nanoparticles and RNA Binding Compounds

Nanoparticles have received considerable attention recently, partly because of their potential to carry drugs to target cancer cells, and some formulations specifically target the nucleolus [197,198]. For example, they have been shown to induce protein aggregates blocking transcription, damaging rDNA, or disrupting the nucleolar structure, altering the localization of nucleolar proteins NPM1 and POLR1A (see ref. [12]). Furthermore, RNA-targeting small molecules can be envisaged as a future strategy to disrupt RiBi by blocking functions of rRNA or other nucleolar RNA species [199,200].

RNA binding molecules can be used in visualizing the nucleolus. In detail, the relationship between Pt(II) compounds with the nucleolus has been widely exploited to develop platinum-based probes for cell-based imaging of the nucleoli. A clickable, azide-containing Pt(II) complex was found to localize in nucleoli post-treatment and after fixation of the cells, emitting a strong fluorescence signal [201]. Additionally, a water-soluble alkynylplatinum(II) complex was recently developed as a luminescent nucleolar probe [202]. The complex exhibited a novel sensing mechanism involving aggregation with RNA and supramolecular self-assembly. For more information about platinum-acridine hybrid agents, monofunctional planar and nonplanar complexes, nanoparticles, naphthalene diimides [203], and various RNA dyes, see the review by Pickard and Bierbach [204].

## 5. Preclinical and Clinical Applications on Certain Cancer Types

How can the accumulating knowledge on the effects of RiBi inhibition be implemented to power its potential for further clinical development and application in cancer therapy? In the era of targeted therapies and personalized medicine, patient stratification is imperative to guide the development of therapy prediction and optimize clinical efficiency. While the mechanistic understanding and clinical application of RiBi inhibition remain to be further explored, evidence suggests that molecular and genetic associations of predictive value regarding response to therapy are indeed emerging. Thus, RiBi rate is a determinant factor for cancer cell sensitivity to RiBi inhibition. One of the methods employed to indirectly assess rDNA transcriptional activity in tumor tissue sections is AgNOR staining, by measuring the size and number of NORs. It is considered a rather powerful survival predictor in many cancers and has been used for patient stratification into low- and high-risk groups in multiple myeloma, pharyngeal, and prostate carcinoma, among others [205]. Pol I activity and POLR1A abundance have also been considered as promising biomarkers for the identification of cancers sensitive to Pol I inhibition, leading to the development of an rRNA transcription assay applicable to human cancer specimens [156,206]. Finally, rDNA chromatin activity status has been investigated as a biomarker, showing an association of the proportion of active to inactive rDNA repeats with ovarian cancer cell sensitivity to CX-5461 [207].

Besides the phenotypic changes in Pol I activity or nucleolar size, deregulated oncogenes and tumor suppressors, and activation of signaling pathways associated with RiBi upregulation, constitute key factors influencing cancer cell susceptibility to RiBi inhibition. The p53 status is a determinant factor for cancer cell response to RiBi-inhibitory agents, with p53´s activation being one key mechanism of cytotoxicity. Notably, RiBi rates were found to be directly related to the level of p53 stabilization [208], whereby in cells characterized by high RiBi rates, inhibition triggered a greater degree of p53 stabilization and expression of pro-apoptotic genes. Several studies have shown that Pol I inhibition was more effective in cells harboring wt p53 [10]. On the other hand, p53-independent effects are also observed with compounds such as BMH-21 [157], suggesting that some patients with non-functional p53 may benefit from RiBi inhibition as well.

The RB protein also affects cancer cell sensitivity to RiBi blockade even in the absence of functional p53 [209]. Cells with functional p53 and RB loss have been shown to display increased sensitivity to RiBi inhibitors, including ActD [210]. RB loss was found to be a predictor of good clinical outcomes in a cohort of breast cancer patients receiving chemotherapy with the RiBi-interfering agents 5-FU, methotrexate, and cyclophosphamide [211]. An additional common event in many cancer types that results in elevated RiBi rates and is predicted to sensitize cancer cells to RiBi inhibitors is the loss of PTEN or p14/p19ARF, both negative regulators of Pol I activity. However, further data is needed to establish the anticipated dependencies and validate the power of these tumor suppressors in predicting clinical outcomes of RiBi-targeting therapy.

RiBi inhibition might benefit tumors characterized by up-regulated RiBi consequent to activation or overexpression of positive regulators. MYC-driven malignancies have been shown to respond well to RiBi inhibitors, and its expression levels represent a potential biomarker that could predict therapeutic outcomes [7]. Commonly hyperactivated in cancer, the RTK (Receptor Tyrosine Kinase) growth signalling pathways converge to the downstream RAS-MAPK and PI3K-mTOR pathways, interacting with the Pol I machinery components and upregulating rRNA synthesis [4]. RTK hyperactivation could therefore render cells sensitive to Pol I inhibition and be used as a therapeutic response indicator. The regulatory connection of the nucleolus with growth signalling pathways and oncogenes offers several opportunities for designing tailored RiBi-targeting therapies in specific molecular signatures. However, the hierarchy and importance of the factors mentioned above in determining clinical outcomes remain largely unexplored.

By employing bioinformatics tools, several studies have highlighted the addiction of certain cancer types based on their molecular signatures to highly active Pol I, providing a foundation for more precise, detailed molecular characterization of the tumors and rational patient selection to ensure maximal benefit from RiBi inhibition. For example, Bruno et al., who pinpointed the platinum drug oxaliplatin as a RiBi inhibitor, aimed to elucidate the observed sensitivity of colorectal cancers to oxaliplatin on the basis of the molecular cancer signatures [153]. By comparing gene expression patterns across all available colorectal cancer samples obtained from The Cancer Genome Atlas (TCGA), it was concluded that this cancer is characterized by translation addiction, as indicated by the upregulation of genes and enrichment of pathways related to the ‘ribosome’. This could explain the higher sensitivity to RiBi blockade. Additionally, these authors identified a correlation between *APC* expression and sensitivity to oxaliplatin, which was also shown in breast and lung cancer tumor samples.

A similar approach was employed for high-risk neuroblastoma, a cancer type characterized by genetic amplification and overexpression of n-MYC and, in some cases, of c-MYC oncogenes [212]. An unsupervised clustering analysis performed on several neuroblastoma patient cohorts showed that high expression of *MYCN* correlates with advanced-stage disease and upregulation of genes involved in RiBi. To further explore this observation, the authors treated neuroblastoma cell lines and xenografts with quarfloxin or CX-5461 and reported cytotoxic effects and tumor size reduction [212]. Reduced n-MYC and Pol I activity was observed; however, since both compounds have been shown to stabilize G4s present at oncogenic promoters such as *c*-*MYC*, the observed Pol I downregulation could be an indirect consequence of MYC downregulation, indicating the possibility that G4 stabilization was the primary mechanism resulting in cancer cell death. Prostate cancer has also been shown to exhibit upregulated Pol I activity and increased rRNA levels, considered to relate to c-MYC overexpression. Based on these observations, BMH-21 was tested in metastatic cancer cell lines, showing effective growth inhibition in a p53-independent manner. BMH-21 reduced tumor size and the Ki67 proliferation marker in an enzalutamide-resistant xenograft model and an aggressive genetically modified mouse prostate cancer model [213].

Regardless of the potential of RiBi as a therapeutic target, cancers are often characterized by inherent resistance to monotherapies, highlighting the need for rationally designed combinatorial regimens. To this end, several efforts have been made to identify drug classes that synergize with RiBi inhibitors in attenuating cancer cell growth. CX-5461 synergizes with PARP inhibition, enhancing replication stress in homologous recombination (HR) DNA repair-deficient ovarian cancer cells [214]. In another study, TOP1 inhibition cooperated with CX-5461 in HR-proficient high-grade serous carcinomas [215]. Promising results in various cancer models have been obtained by combinations with radiation, a p53 activator, and mRNA translation modulators [216,217,218,219]. Overall, combinatorial RiBi inhibition has the potential to enhance the therapeutic response and is an area of ongoing research. Taken together, these experimental studies strengthen the concept of Pol I inhibition as a targetable vulnerability. Further characterization, development, and clinical application of RiBi inhibitors will hopefully enable patient stratification and personalized treatment options.

## 6. Conclusions

From one point of view, one may wonder why we spend time and research resources on RNA Pol I inhibitors when several cancer drugs used for decades are exhibiting such effects, and quite effectively. However, the increasing number of mechanistic studies have shown that most of these drugs exhibit dose-dependent pleiotropic effects and are rather non-specific. They have chemical structures associated with certain toxicity mechanisms, and today, several compounds would most likely not be considered in the drug development process. The search for novel or improved RiBi inhibitors therefore continues. In parallel, there are surprising recent discoveries being made as to the mechanism of action of classical molecules such as ActD, 5-FU, and oxaliplatin. Continued efforts are aimed at developing more specific RiBi inhibitors than the ones available today, as no currently available compound is truly a Pol I-specific inhibitor.

So, what does the future hold? Drug discovery is continuously being advanced by the rapid progress in various microscopy and cell painting methods, coupled with in silico screening approaches [220,221,222]. Novel computational, image analytics and data mining methods have been developed and enabled the high throughput detection of phenotypical changes of the nucleolus, such as changes in nucleolar numbers or in the synthesis of rRNA, unleashing an extended chemical space exploration, and thus, the discovery of unique pharmacophores [137,223,224,225]. The use of such tools and approaches is expected to increase dramatically in the future, complementing the conventional screening methods, as conducted over the past decades. Additional information may rapidly emerge from genetic screenings that transform various pathways into mechanistic information, aiding in drug classification. Integrated multi-omics already being used for drug response profiling are also expected to play an integral role in mechanistic characterization and bring RiBi inhibition even closer to the clinic [121,226,227]. All of these tools will also aid in drug repurposing. We may hopefully see re-vitalized rational drug design since there are many beautiful ribosome structures now available [228,229]. To succeed, we must also increase our understanding of the complex process that RiBi constitutes. Much remains to be discovered about the nucleolus, RiBi, and the heterogeneous ribosomes of cancer cells. We should then keep in mind the concept of the multifunctional nucleolus. The effects of the drugs are not limited to building ribosomes; the destruction of the nucleolus is likely to significantly impact many cellular functions.

## Abbreviations

ActD	Actinomycin D
AAA-ATPases	*ATPases* Associated with diverse cellular Activities
CDK	Cyclin Dependent Kinase
CRC	Colorectal Cancer
DDR	DNA Damage Response
DHODH	Dihydroorotate Dehydrogenase
EMT	Epithelial to Mesenchymal Transition
hERG	human Ether-a-go-go Related gene
HMG	High-Mobility Group
IC_50_	Half Maximal Inhibitory Concentration
IMPDH	Inosine-5’-monophosphate dehydrogenase
IRBC	Impaired Ribosome Biogenesis Checkpoint
MAPK	Mitogen-activated protein kinase
MDM2	Mouse Double Minute 2
mTOR	Mechanistic Target of Rapamycin
PIC	Pre-Initiation Complex
Pol I	RNA Polymerase I
Pol II	RNA Polymerase II
PTEN	Phosphatase and Tensin Homolog
RB	Retinoblastoma protein
rDNA	Ribosomal DNA
RiBi	Ribosome Biogenesis
RP	Ribosomal Protein
rRNA	Ribosomal RNA
RTK	Receptor Tyrosine Kinase
SL1	Selectivity Factor 1
TIF-I	Transcription Initiation Factor I
TOP1/TOP2	Topoisomerase 1 and 2
UBF	upstream binding factor
5-FU	5-fluorouracil
5S RNP	5S ribonucleoprotein
